# Potential of Quercetin as a Promising Therapeutic Agent Against Type 2 Diabetes

**DOI:** 10.3390/molecules30153096

**Published:** 2025-07-24

**Authors:** Przemysław Niziński, Anna Hawrył, Paweł Polak, Adrianna Kondracka, Tomasz Oniszczuk, Jakub Soja, Mirosław Hawrył, Anna Oniszczuk

**Affiliations:** 1 Department of Pharmacology, Medical University of Lublin, Radziwiłłowska 11, 20-080 Lublin, Poland; przemyslawnizinski@umlub.pl; 2 Department of Inorganic Chemistry, Medical University of Lublin, Chodźki 4a, 20-093 Lublin, Poland; anna.hawryl@umlub.pl (A.H.); miroslaw.hawryl@umlub.pl (M.H.); 3 Department of Orthopedics and Treumatology, Provincial Specialist Hospital in Biała Podlaska, Terebelska 57, 21-500 Biała Podlaska, Poland; pawel.polak@szpitalbp.pl; 4 Department of Obstetrics and Pathology of Pregnancy, Medical University of Lublin, 20-081 Lublin, Poland; adrianna.kondracka@umlub.pl; 5 Department of Thermal Technology and Food Process Engineering, University of Life Sciences in Lublin, Głęboka 31, 20-612 Lublin, Poland; tomasz.oniszczuk@up.lublin.pl (T.O.); jakub.soja@up.lublin.pl (J.S.)

**Keywords:** quercetin, polyphenols, nutraceuticals, functional food, oxidative stress, antioxidants

## Abstract

Quercetin (QE) is a naturally occurring flavonoid found in many fruits, vegetables, and other plant-based foods. It is recognized for its diverse pharmacological activities. Among its many therapeutic potentials, its antidiabetic properties are of particular interest due to the growing worldwide prevalence of diabetes mellitus. QE improves glycemic control by enhancing insulin sensitivity, stimulating glucose uptake, and preserving pancreatic beta cell function. These effects are mediated by the modulation of key molecular pathways, including AMPK, PI3K/Akt, and Nrf2/ARE, as well as by the suppression of oxidative stress and pro-inflammatory cytokines, such as TNF-α and IL-6. Furthermore, QE mitigates the progression of diabetic complications such as nephropathy, retinopathy, and vascular dysfunction, reducing lipid peroxidation and protecting endothelial function. However, the clinical application of quercetin is limited by its low water solubility, poor bioavailability, and extensive phase II metabolism. Advances in formulation strategies, including the use of nanocarriers, co-crystals, and phospholipid complexes, have shown promise in improving its pharmacokinetics. This review elucidates the mechanistic basis of QE quercetin antidiabetic action and discusses strategies to enhance its therapeutic potential in clinical settings.

## 1. Introduction

Diabetes is a chronic metabolic disease that has been categorized by the American Diabetes Association (ADA) into four major types based on their distinct pathogenesis: type 1 diabetes, type 2 diabetes, diabetes due to other causes, and gestational diabetes. It has been confirmed that type 2 diabetes accounts for 90–95% of all cases [1]. Type 2 diabetes is caused by defective insulin secretion by pancreatic cells and the inability of insulin-sensitive tissues to respond appropriately to insulin. Insulin synthesis, release, and activity are essential elements in glucose homeostasis. These processes are regulated by molecular mechanisms, any disruption of which can lead to an imbalance in the metabolic balance responsible for the development of the disease [2]. According to the literature, type 2 diabetes occurs mainly in adults, but in recent years it has also been increasing in children and adolescents, which is closely related to obesity, a lack of physical activity, and improper nutrition. The occurrence of type 2 diabetes in a given person is associated with several risk factors and interactions between them. Such risk factors include, among others, lifestyle, general health, genetic, psychosocial, and demographic factors. People with depression, hypertension, dyslipidemia, circulatory system diseases, as well as those with sleep problems, low physical activity, and who are elderly, obese, and smoking are at high risk of disease [3]. There are many possible actions to prevent or reduce the risk of developing type 2 diabetes, including the following: preventing and treating obesity, including pre-pregnancy obesity, preventing metabolic dysfunction associated with fatty liver disease (diet), high physical activity, and avoiding stress. If it is no longer possible to prevent this disease, efforts should be made to detect it early and start treatment [4]. The very high incidence of type 2 diabetes and the high mortality rate of patients with this disease have led researchers to search for innovative preventive and therapeutic solutions.

The preferred and most widely used first-line drug for the treatment of type 2 diabetes worldwide is metformin, an oral drug that lowers blood glucose levels by several mechanisms, including the inhibition of hepatic gluconeogenesis (by activating AMP-activated protein kinase), reduction in lipogenic enzyme expression, and inhibition of cellular respiration (by inhibiting mitochondrial complex I). Among the next group of antidiabetic drugs are sulfonylureas, glinides, or meglitinides, which increase insulin release from pancreatic beta cells. Another group of drugs is Thiazolidinediones, which cause activation of the gamma isoform of the peroxisome proliferator-activated receptor (PPAR gamma), a nuclear receptor. Alpha-glucosidase inhibitors (AGIs) are also drugs used in the treatment of type 2 diabetes. They can prevent the increase in blood glucose levels by inhibiting the enzymatic digestion of carbohydrates in the intestinal lumen. Next, there are dipeptidyl peptidase 4 (DPP-4) inhibitors, a ubiquitous enzyme that acts on incretin hormones, mainly GLP-1 (glucagon-like peptide-1) and GIP (gastric inhibitory peptide), which maintain glucose homeostasis by increasing insulin secretion and decreasing glucagon secretion. Another group of drugs is sodium–glucose cotransporter-2 (SGLT2) inhibitors, which lower blood glucose levels through glycosuria and natriuresis initiated by the inhibition of glucose reabsorption in the proximal renal tubule. In contrast, glucagon-like peptide-1 receptor agonists (GLP-1 RAs) increase glucose-dependent insulin secretion and reduce inappropriate glucagon secretion, delay gastric emptying, and increase satiety. However, many of these drugs are expensive and have many side effects [5].

**Figure 1:**
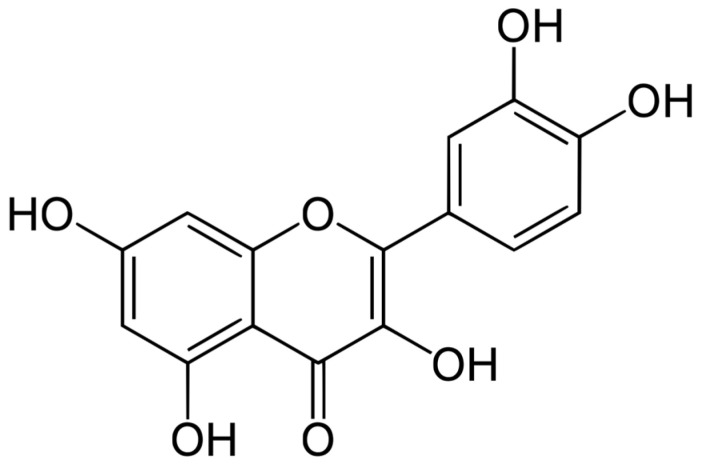
Chemical formula of quercetin.

Therefore, herbal medicines can be an alternative and/or complement to the treatment of type 2 diabetes. These substances are certainly cheaper, as well as more available, and also have minor side effects. According to the literature, many plant substances with antioxidant properties have antidiabetic activity, including polyphenolic compounds from the flavonoid group [6]. The flavonoid group includes quercetin (3,3′,4′,5,7 pentahydroxyflavone), which is commonly found in vegetables, fruits, wine, and tea [7]. This substance has strong antioxidant [8,9] properties, as well as antibacterial [9,10,11], immunostimulating [12,13], anticancer [14,15,16], anti-inflammatory [17,18], anti-allergic [19,20] and antidiabetic properties [21,22]. The chemical structure of QE is shown in Figure 1.

In recent years, there has been an increase in the number of studies focusing on the potential use of quercetin in the treatment of diabetes and the prevention of its complications. It is indicated in scientific reports that the incidence of both diabetes and its metabolic complications can be significantly reduced by the use of quercetin as a therapeutic agent. A plethora of preclinical studies have delineated the mechanisms by which quercetin exerts its antidiabetic effects. It has been demonstrated that QE possesses the capacity to reduce blood glucose levels through a number of complementary mechanisms.

**Figure 2:**
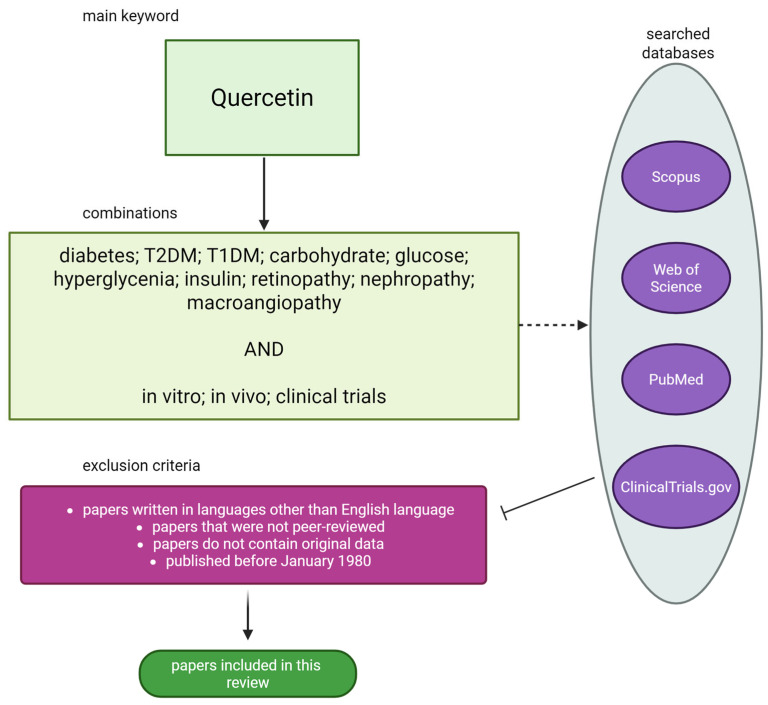
Flowchart of data collection. Created in BioRender^®^.

The present article constitutes a review of the extant literature pertaining to the therapeutic effects of QE in diabetes and its complications. In addition, the molecular mechanism by which QE exerts its therapeutic effects on these diseases is discussed, given the paucity of studies addressing this issue in a comprehensive manner. This will facilitate a more profound comprehension of the therapeutic properties of QE and establish a point of reference for subsequent research and applications of QE. Notwithstanding the encouraging biological characteristics exhibited by QE, its clinical implementation remains constrained by its limited bioavailability, a subject that will be addressed in the ensuing article. The literature search was conducted using the PubMed, Scopus, Web of Science, and ClinicalTrials.gov databases. The following queries were used: ‘Quercetin’ and ‘diabetes’; ‘T2DM’; ‘T1DM’; ‘carbohydrate’; ‘glucose’; ‘hyperglycemia’; ‘insulin’; ‘retinopathy’; ‘nephropathy’; ‘macroangiopathy’. All of the above combinations were also supplied with the terms ‘in vitro’, ‘in vivo’, or ‘clinical trials’. Papers ranging from 1980 to 2025 were included. To qualify for inclusion in this review, papers had to meet the following criteria: contain original data; have been independently reviewed; be written in English; and have been published after January 1980. Figure 2 shows a flowchart illustrating the data collection process for this review.

## 2. Diabetes and Role of Oxidative Stress

Oxidative stress, an imbalance between the production of reactive oxygen species and the body’s antioxidant capacity to remove them, is one of the main factors influencing the development and complications of type 2 diabetes. In type 2 diabetes, chronic hyperglycemia and hyperlipemia cause excessive production of reactive oxygen species, which automatically increases the phenomenon of oxidative stress. The mechanism of this process is related to mitochondrial dysfunction (increased mitochondrial respiration), as well as the increased activity of nicotinamide adenine dinucleotide phosphate oxidase or the unnatural excessive growth of some prooxidant processes [23,24]. High overproduction of reactive oxygen species can modify the structure of proteins, lipids, or nucleic acids, and, consequently, their improper functioning [25]. In addition, it may lead to impaired insulin production (impaired β-cell function), increased insulin resistance, the disruption of glucose metabolism, or the maintenance of hyperglycemic memory. Excessive production of reactive oxygen species caused by hyperglycemia also affects the formation of micro- and macrovascular complications of diabetes, causing systemic inflammation and general organ dysfunction [24,25]. Most often, blood vessels are damaged, and the proper functioning of the endothelium is impaired, which contributes to many vascular complications in type 2 diabetes, such as retinopathy, nephropathy, and cardiovascular diseases. Excessive production of active oxygen species negatively affects the functioning of many tissues and organs (eyes, kidneys, and nerves) and is the cause of retinopathy, diabetic nephropathy, and neuropathy [23].

## 3. Quercetin as a Natural Antioxidant: Sources and Biological Activity

### 3.1. Sources of Quercetin

Flavonoids are a diverse group of polyphenolic compounds that are widely distributed throughout the plant kingdom. They are commonly found in fruits, vegetables, flowers, leaves, or seeds. The major subclasses are flavonols, flavones, flavanones, flavanols, anthocyanins, and isoflavones. In plants, flavonoids play essential roles in growth and development, pigmentation, UV protection, and defense against pathogens. They also act as signaling molecules in plant–microbe interactions and contribute to the plant antioxidant defense system by scavenging reactive oxygen species [26,27]. Quercetin is one of the most abundant secondary plant metabolites, belongs to the subclass of flavonols, and, as mentioned earlier, has very strong antioxidant properties. Maintaining oxidative balance may be due to direct antioxidation and scavenging free radicals or via influencing enzymatic or signal transduction pathways [28]. There are two general species of QE: an aglycone, rarely occurring in natural sources, and glycosides, which consist of an aglycone and a sugar moiety connected by a glycosidic bond, while 3-O-glycosides are found to be most abundant [29,30]. The richest in QE and its glycoside natural products are herbs and spices such as capers, lovage, and dill; vegetables such as onion and pepper; and fruits as cranberries and lingonberries [31]. The most prominent sources of dietary QE in the form of both aglycones and glycosides are summarized in Table 1.

### 3.2. Anticancer Properties

Quercetin has attracted considerable attention due to its wide-ranging anticancer properties. Studies in both vitro and vivo demonstrate that QE inhibits proliferation, induces apoptosis, and suppresses metastasis across various cancer types [32]. These effects are mediated through interaction with multiple signaling pathways, gene expression regulators, and epigenetic modulators [15]. At a molecular level, QE primarily exerts its pro-apoptotic and antiproliferative activity through the p53, PI3K/Akt/mTOR, MAPK, and Wnt/β-catenin signaling cascades [33]. In the PI3K/Akt pathway, quercetin inhibits the phosphorylation of PI3K and its downstream effectors, such as mTOR and p70S6K. A study using prostate cancer cells PC-3 and DU145 demonstrated that the expression of phosphorylated Akt and mTOR is reduced by both QE alone and docetaxel combined with QE, resulting in growth inhibition and enhanced apoptosis [34,35]. Quercetin has also been reported to interfere with the Wnt/β-catenin signaling pathway, which is involved in various cellular processes, including cell growth, differentiation, and migration. In SW480 colon cancer cells, QE decreased β-catenin nuclear translocation and downregulated target genes such as c-Myc and cyclin D1, thereby reducing cell proliferation [36]. In breast cancer cell lines MCF-7 and MDA-MB-231, quercetin induces autophagy processes by inhibiting the Akt-mTOR pathway [37]. Also, in vivo studies in mice models of MCF-7 and CT-26 tumors resulted in a significant reduction in tumor volume after intraperitoneal QE administration in the dose range 100–200 mg/kg [38]. In addition, it has been found to target key processes involved in tumor angiogenesis and metastasis [39]. It downregulates the expression of the vascular endothelial growth factor (VEGF), which is a central mediator of angiogenesis. This impairs the formation of new blood vessels, which are required for tumor growth and nutrient supply [40,41]. It also suppresses the activity and expression of matrix metalloproteinases (MMPs), particularly MMP-2 and MMP-9, which play a key role in extracellular matrix degradation and cancer cell invasion [42]. By inhibiting VEGF-mediated angiogenic signaling and MMP-driven metastatic processes, QE exerts dual anti-angiogenic and anti-metastatic effects, significantly contributing to its anticancer potential.

### 3.3. Anti-Microbial Properties

Polyphenols including QE exhibit broad-spectrum antimicrobial activity against bacteria, fungi, and viruses. This is attributed to multiple molecular mechanisms that interfere with microbial viability, virulence, and host–pathogen interactions. Disrupting bacterial membrane integrity is one of the main ways that quercetin displays its antibacterial properties. This results in increased permeability, the leakage of intracellular contents, and cell lysis [43]. This membrane-disrupting effect is accompanied by the inhibition of nucleic acid synthesis, including the suppression of DNA gyrase and topoisomerase IV enzymes, which are essential for bacterial DNA replication and transcription [44]. Furthermore, QE influences bacterial energy metabolism by interfering with ATP synthesis and possibly altering oxidative stress responses [45,46]. It also modulates quorum-sensing pathways, thereby reducing the expression of virulence factors and inhibiting biofilm formation, which is a critical mechanism in bacterial resistance and chronic infection [47]. In Gram-negative pathogens such as *Pseudomonas aeruginosa*, *Escherichia coli*, *Chromobacterium violaceum*, *Serratia marcescens*, and Gram-positive pathogens such as *Listeria monocytogenes*, QE has been shown to downregulate genes responsible for biofilm development, flagellar motility, and secretion systems [11,48,49,50]. In fungal species, particularly *Candida albicans*, QE impairs cell wall synthesis, suppresses hyphal transition, and inhibits the efflux pumps involved in antifungal resistance [51,52]. Its antiviral effects are mainly attributed to interference with viral entry, replication, and protein assembly. For instance, quercetin exhibits inhibitory activity against influenza, herpes simplex virus, and SARS-CoV-2 by targeting viral proteases and preventing host–virus interactions [53].

### 3.4. Anti-Allergenic Potential

Quercetin exhibits potent anti-allergic properties by modulating multiple immune and inflammatory pathways implicated in immediate and delayed-type hypersensitivity reactions [54]. At a cellular level, QE inhibits the degranulation of mast cells and the release of allergic mediators, such as histamine, tryptase, prostaglandin D2 (PGD2), and leukotrienes. This occurs primarily by suppressing the high-affinity IgE receptor (FcεRI) signaling cascade and by inhibiting intracellular calcium influx in sensitized mast cells and basophils [20,55]. In vitro studies using human mast cells HMC-1, RBL-2H3, and LAD2 cells have shown that QE significantly reduces the secretion of IL-1β, IL-4, IL-5, IL-6, IL-8, and TNF-α and downregulates the phosphorylation of key upstream kinases in allergic signal transduction, such as Lyn, Syk, and Akt [19,56,57,58].

In vivo, QE has been shown to reduce allergic airway inflammation and hyperresponsiveness in asthma models in mice by decreasing eosinophil infiltration, goblet cell hyperplasia, and mucin production. These effects are accompanied by decreased serum levels of total IgE and ovalbumin-specific IgE, as well as the suppression of Th2 cytokines (IL-4, IL-5, and IL-13) and epithelial-derived proallergic cytokines, such as IL-33 and TSLP [20]. In a mouse model of atopic dermatitis, the oral administration of quercetin improved skin lesions, reduced epidermal thickening, and lowered the local expression of IL-1β and TNF-α, which further supports its anti-inflammatory and immunomodulatory effects [59]. Also, topical administration of QE in atopic dermatitis mouse models has shown promising results, decreasing the levels of pro-inflammatory cytokines as well as less erosion and epidermal hyperplasia in skin lesions [60,61]. Furthermore, QE appears to restore the balance between Th1 and Th2 cells by promoting IFN-γ production and inhibiting GATA3 expression, thereby dampening the Th2-biased immune responses typically observed in allergic conditions [55].

### 3.5. Anti-Aging and Senolytic Properties

The anti-aging effects of QE are well documented and have been demonstrated in both in vitro and in vivo experimental models through its antioxidant, anti-inflammatory, and senolytic actions. In human embryonic fibroblasts (HFL-1), QE reversed several features of cellular senescence, including enhancing proteasome function, restoring youthful cell morphology, and reducing senescence-associated β-galactosidase (SA-β-gal) activity [62]. These effects were associated with the activation of the Nrf2 pathway and increased the expression of antioxidant enzymes, such as HO-1 and γ-glutamylcysteine synthetase [62,63]. Similarly, in quercetin-treated human dermal fibroblasts (HDFs), a dose-dependent reduction in reactive oxygen species (ROS) and downregulation of p53 and p21 expression resulted in delayed replicative senescence [64]. In addition to its effects on cellular aging, QE exhibits senolytic activity by selectively inducing apoptosis in senescent cells. Xu et al. [65] demonstrated that a single dose of QE combined with dasatinib, a tyrosine kinase inhibitor, reduced the burden of senescent cells in aged or irradiated mice. This resulted in improved cardiac function, reduced adipose tissue inflammation, and increased physical performance, including treadmill endurance and grip strength. Furthermore, repeated treatment over several months increased median and maximal lifespan in naturally aged mice [65]. Beyond fibroblast and mesenchymal stromal cell models, QE protects against age-related mitochondrial dysfunction [66,67]. In aged mice, quercetin administration improved skeletal muscle mitochondrial biogenesis via activation of the SIRT1–PGC-1α axis, reducing inflammatory cytokines, including IL-6 and TNF-α, in serum and muscle tissue [68,69]. In a dose-dependent manner, QE significantly extended the lifespan of *Simocephalus vetulus* by enhancing the activity of antioxidant enzymes (SOD, CAT, and GSH-Px) and reducing oxidative stress markers. Proteomic analysis revealed the upregulation of proteins involved in redox regulation, energy metabolism, and protein folding, as well as the activation of longevity-associated pathways such as PI3K-Akt and FoxO [70]. As the promising results of preclinical studies of QE combined with dasatinib, pilot clinical trials have been conducted targeting idiopathic pulmonary fibrosis [71] or cognition and mobility in older adults [72]; furthermore, a number of clinical trials are currently ongoing [73].

### 3.6. Cardiovascular Disease Treatment

Quercetin exhibits potent cardioprotective effects, which are mediated by its antioxidant, anti-inflammatory, lipid-lowering, vasodilatory, and antithrombotic properties. This makes QE a promising candidate for the prevention and management of cardiovascular diseases (CVDs) [74]. One of its key mechanisms involves enhancing endothelial function by increasing nitric oxide (NO) production. It upregulates endothelial nitric oxide synthase (eNOS) while simultaneously downregulating NADPH oxidase-derived ROS, thereby restoring NO bioavailability and improving vasodilation [75]. These effects contribute to lowering blood pressure, as demonstrated in both animal models and human trials [76]. A meta-analysis of randomized controlled trials reported that quercetin supplementation (≥500 mg/day) significantly reduced both systolic and diastolic blood pressure in hypertensive patients [77]. QE also exhibits anti-atherosclerotic activity by attenuating lipid accumulation and endothelial inflammation [78]. It inhibits LDL oxidation and reduces the expression of vascular adhesion molecules, such as VCAM-1 and ICAM-1, which are crucial for monocyte adhesion and foam cell formation in the early stages of atherogenesis [79]. In apolipoprotein E-deficient mice, QE administration the reduced aortic plaque area and decreased serum levels of total cholesterol, LDL-C, and triglycerides [80]. At a molecular level, QE modulates inflammatory pathways such as NF-κB, MAPK, and JAK/STAT, thereby downregulating pro-inflammatory cytokines including IL-6, TNF-α, and MCP-1 in vascular tissues [81]. In a study by Albadrani et al. [82], it was demonstrated that quercetin has cardioprotective effects in rats with isoproterenol-induced myocardial infarction by activating the JAK2/STAT3 signaling pathway, thus upregulating antioxidant enzymes such as SOD, catalase, and glutathione peroxidase while suppressing iNOS expression and lipid peroxidation. Treatment with QE also preserved myocardial architecture, reduced infarct size, and inhibited apoptotic cell death by modulating Bcl-2/Bax ratios and caspase-3 activity [82]. Furthermore, QE inhibits platelet aggregation and thrombus formation by interfering with calcium signaling and suppressing thromboxane A2 production, thereby reducing the risk of thromboembolic events [83]. As there is a plethora of information regarding the role of QE in CVDs, both preclinical and clinical evidence, readers are referred to a recently published comprehensive review about the potential of quercetin in the prevention and treatment of cardiovascular events [84].

### 3.7. Neuroprotective Properties

Neuroprotection refers to strategies or interventions that preserve the structure and function of neurons by preventing or slowing down injury, degeneration, or death of these cells. Such mechanisms usually target oxidative stress, inflammation, mitochondrial dysfunction, and excitotoxicity in order to maintain neural integrity and support brain homeostasis [85]. Quercetin has been shown to have strong neuroprotective properties by modulating oxidative stress, inflammation, mitochondrial dysfunction, and apoptosis, which are central mechanisms in the pathogenesis of neurodegenerative disorders. As a potent free radical scavenger, QE reduces oxidative stress by directly neutralizing ROS and increasing the expression of endogenous antioxidant enzymes, such as SOD, CAT, and GSH-Px, by activating the Nrf2/ARE signaling pathway [86]. In murine cortical brain tissue cell cultures, QE mitigates H_2_O_2_-induced cytotoxicity and lipid peroxidation, preserving mitochondrial membrane potential and reducing intracellular ROS levels [87]. In studies using PC12 cells, QE restored cell viability, prevented caspase-3 activation, and maintained Bcl-2 expression following oxidative injury, demonstrating its potent anti-apoptotic properties as well as regulating the SIRT1/Nrf2/HO-1 signaling pathway, thus alleviating oxidative stress and ultimately increasing the survival rate of neuronal cells [88,89,90]. In vivo, the administration of quercetin in rodent models of Alzheimer’s disease (AD) and Parkinson’s disease (PD) has revealed promising results. In AD mouse models, chronic quercetin treatment reduced β-amyloid (Aβ) plaque deposition, improved spatial memory performance in the Morris water maze test, and reduced markers of hippocampal oxidative stress. These effects were accompanied by the downregulation of acetylcholinesterase activity, suggesting enhanced cholinergic neurotransmission [86]. In PD models induced by neurotoxins such as 6-hydroxydopamine (6-OHDA) and rotenone, QE preserved dopaminergic neuron density in the substantia nigra and increased striatal dopamine levels, in part by reducing mitochondrial dysfunction and microglial-mediated neuroinflammation [86]. QE inhibits key pro-inflammatory pathways, including NF-κB, MAPKs (ERK, JNK, and p38), and COX-2. This leads to reduced levels of TNF-α, IL-1β, and iNOS in cell and animal models of neuroinflammation [91]. For example, in mice treated with lipopolysaccharide (LPS), QE reduced microglial activation and suppressed the TLR4/NF-κB signaling cascade, hence decreasing the expressions of TNF-α, COX-2, NOS-2, and IL-1β [92]. Furthermore, QE has demonstrated neuroprotective properties in models of ischemic stroke, reducing infarct volume, improving neurological scores, and preserving blood–brain barrier (BBB) integrity, probably by suppressing matrix metalloproteinases and promoting tight junction proteins [93]. An updated review that comprehensively describes recent advancements in the field of neuroprotective effects of QE has been recently published elsewhere [94].

## 4. Bioavailability of Quercetin

Quercetin, despite its strong intrinsic activity and thus high efficacy proven in vitro, presents poor bioavailability. Thus, its efficacy in vivo does not always correspond with preclinical trial results. QE is known as poorly soluble in body fluids; the solubility of QE is about 1 μg/mL, 5.5 μg/mL, and 28.9 in water, gastric, and intestinal fluid, respectively [95]. In general, after oral administration, the bioavailability of QE is about 10% [96]. In comparison to QE aglycone, its glycosides could be characterized with better bioavailability; for instance, quercetin 3-O-β-glucuronide has been shown to be better absorbed than the parent compound in rats [97]. Following oral administration, QE undergoes extensive phase I and phase II metabolism, primarily in the small intestine and liver [98]. This results in the formation of several conjugated metabolites. In the intestinal epithelium, quercetin glycosides are hydrolyzed by lactase-phlorizin hydrolase (LPH) or cytosolic β-glucosidases, releasing the aglycone that is then absorbed by enterocytes [99,100]. Once inside the cell, QE undergoes rapid conjugation through phase II enzymes: uridine-5′-diphospho-glucuronosyltransferases (UGTs) catalyze glucuronidation, sulfotransferases (SULTs) mediate sulphation, and catechol-O-methyltransferase (COMT) performs methylation of the hydroxyl groups. The main circulating forms in plasma are quercetin-3-O-glucuronide, quercetin-3’-sulfate, and isorhamnetin [101]. Concentrations of these metabolites are largely dependent on interindividual variation in enzyme expression and gut microbiota composition [102]. These conjugated metabolites retain partial biological activity and may serve as a reservoir for deconjugated, active QE in target tissues. After absorption, quercetin metabolites are transported to the liver via the portal vein, where they undergo further conjugation before being distributed throughout the body. Studies have shown that aglycone QE is rarely detected in plasma and that its bioactivity in vivo is primarily mediated by its metabolites, which may be selectively deconjugated at sites of inflammation or oxidative stress. Additionally, some quercetin conjugates undergo enterohepatic recirculation, contributing to a prolonged systemic presence. The metabolites are ultimately excreted in urine and bile, with glucuronides as the predominant urinary forms [103]. Enhancing the bioavailability of quercetin becomes a crucial step in the augmentation of its therapeutic potential. So far, a number of strategies have been proposed in order to improve the bioavailability of orally administered QE. Strategies such as nanoformulation, co-crystallization, and encapsulation have shown promise in enhancing solubility and systemic exposure [104]. Notably, the co-crystallization of quercetin with nicotinamide increased plasma concentration by 392%, and amorphous solid dispersions improved solubility and peak plasma levels [105]. Also, nanoemulsions, liposomes, and hydrogel beads have shown promising results in enhancing quercetin bioavailability. For instance, nanoemulsions stabilized with rice bran protein increased QE bioavailability ninefold compared to the unencapsulated form [106]. Furthermore, dietary fat, prebiotics such as fructooligosaccharides, and food matrices such as muffins or emulsified foods can significantly enhance intestinal absorption by promoting micellar solubilization and stability in gastrointestinal fluids [96]. These findings emphasize the importance of delivery system design and food matrix interactions in overcoming quercetin’s bioavailability barriers for its effective clinical application.

## 5. Potential Antidiabetic Effects of Quercetin

Many experiments have focused on the hypoglycemic properties of quercetin, using various animal models. In experimental diabetes models, such as rats with streptozotocin-induced diabetes, alloxan, and a combination of nicotinamide and streptozotocin, oral administration of QE at various doses led to significant reductions in blood glucose levels, as well as reductions in total hemoglobin and glycated hemoglobin. Moreover, a protective effect against body weight was also observed—quercetin prevented weight loss in animals with type 1 diabetes. Many QE dosing regimens have been described in the scientific literature, including the following: 100 mg/kg for 49 days [107], 50 and 80 mg/kg for 45 days [108], 100 and 200 mg/kg for 6 weeks [109], 25, 50, and 75 mg/kg for 28 days [110], 30 mg/kg body weight for 2 weeks [111], 10 and 15 mg/kg for 2 weeks [112], and 8 weeks [113]. In addition, a beneficial effect was also shown with a diet enriched with QE at a concentration of 0.5%. Its inclusion in the diet of rats with streptozotocin-induced diabetes resulted in a significant reduction in fasting blood glucose levels. Moreover, a reduction in urinary glucose levels and a decrease in total urinary excretion volume were observed, indicating improved glycemic control and better renal function in diabetes [107]. Overall, oral administration of QE in the dose range of 15 to 100 mg/kg body weight for a period of 14 to 70 days showed marked hypoglycemic effects. The lowering of blood glucose levels was associated with a number of beneficial biological mechanisms, including the restoration and regeneration of pancreatic islets, an increase in serum insulin levels, and the promotion of insulin secretion by pancreatic β cells. Such actions indicate quercetin’s potential ability to partially reverse functional damage to the pancreas under diabetic conditions [107,108,109,110,111,112,113].

In animal models with high-fat diet-induced insulin resistance [114], as well as in studies in mice with type 2 diabetes, such as C57BL/KsJ-db/db strains [115], chronic high-fat intake was found to lead to reduced glucose transport to skeletal muscle. In addition, impaired insulin secretion in response to glucose stimulation and accelerated development of insulin resistance were observed. These adverse changes in glucose metabolism are important pathogenetic factors in type 2 diabetes.

The mechanism of quercetin’s hypoglycemic effect is complex and involves multiple molecular and metabolic processes. One of the key mechanisms is the improvement of insulin signaling—QE increases the expression and phosphorylation of insulin receptor, insulin receptor substrate, and GLUT-type glucose transporters, resulting in improved glucose uptake by cells. In addition, QE can increase tissue sensitivity to insulin, stimulate glycogen synthesis in the liver and muscle, and inhibit the activity of the enzyme α-glucosidase, responsible for the breakdown of carbohydrates in the gastrointestinal tract. At the same time, it shows potential in alleviating insulin resistance, making it a promising ingredient in adjunctive therapy for diabetes and metabolic syndrome [116]. In the case of streptozotocin-induced diabetes, a chemical compound that shows selective toxicity to pancreatic islet β-cells, QE has shown significant protective effects. β-cells, responsible for insulin synthesis and secretion, are destroyed under the influence of streptozotocin, leading to a significant decrease in insulin levels and, consequently, hyperglycemia. Administration of QE in such models not only prevented further loss of β-cells, but also promoted their preservation in terms of number, structure, and function. This enabled the maintenance of relatively stable blood insulin levels and effective glucose lowering, demonstrating the potential role of QE in the prevention and treatment of type 1 diabetes [111].

**Figure 3:**
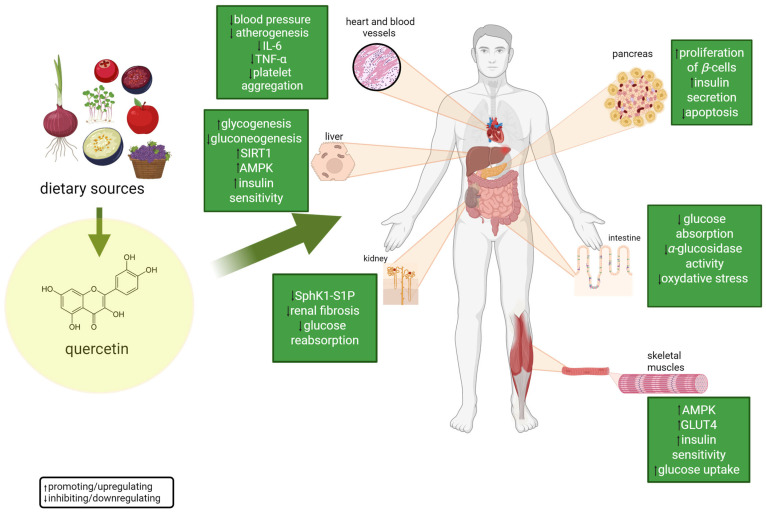
An overview of quercetin biological activity in maintaining normal serum glucose levels. An abbreviation list is given at the end of this article. For further explanations, please see the text below. Created in BioRender^®^.

Quercetin showed similar protective properties in models of alloxan-induced diabetes, another chemical compound used experimentally to induce β-cell damage. In this case, the pathogenetic mechanism mainly involves the induction of oxidative stress, which leads to apoptosis and pancreatic islet cell dysfunction [113]. QE, due to its strong antioxidant properties, reduced the level of ROS, protected cell membranes, and promoted mitochondrial defense mechanisms. As a result, it reduced the breakdown of β-cells, promoted their regeneration, and increased their ability to secrete insulin. This action translated into improving the function of the entire pancreatic islands and counteracting the progression of metabolic disorders characteristic of diabetes. Quercetin not only supported the maintenance of glucose homeostasis but also acted as an agent to prevent further worsening of the diabetic state, making it a potential candidate for therapeutic applications in the treatment of both autoimmune and environmentally induced diabetes [116]. A general overview of QE activity in maintaining proper glucose metabolism is shown in Figure 3.

### 5.1. Promotion of Islet β-Cell Function, Facilitation of Insulin Secretion, and Enhancement in Insulin Sensitivity

Insulin, a hormone that plays a pivotal role in regulating carbohydrate metabolism, is synthesized and secreted by β cells situated within the islets of Langerhans in the pancreas. Dysfunction and viability of β-cells represent a pivotal element in the pathogenesis of both type 1 and type 2 diabetes. The loss of the ability of these cells to produce or secrete insulin results in chronic hyperglycemia and a range of metabolic complications [117].

In recent years, there has been an increasing focus on natural bioactive substances, including flavonoids, as potential adjuncts to diabetes therapy. Of the numerous flavonoids, quercetin is particularly noteworthy, demonstrating robust hypoglycemic properties and the capacity to enhance tissue sensitivity to insulin. This phenomenon can be attributed to a number of factors, including the promotion of the proliferation of pancreatic β-cells, the improvement of glucose metabolism, and the stimulation of insulin secretion [118]. In vitro studies by Youl et al. [119] demonstrated that exposure of pancreatic β-cells from the INS-1 line to 20 mmol/L QE enhanced insulin secretion in response to stimulation with glucose and glibenclamide, a widely prescribed antidiabetic drug. Concurrently, quercetin demonstrated a protective effect against β-cells, safeguarding them from oxidative stress-induced damage. The ERK1/2 kinase signaling pathway, which plays a pivotal role in regulating cell proliferation and survival, was identified as a key component of this mechanism.

The efficacy of QE was also confirmed during in vivo studies. The oral administration of QE at a dose of 120 mg/kg/day for a period of 8 weeks in rats with diabetes mellitus resulted in a significant reduction in both body weight and plasma triglyceride levels. Concurrently, a decline in cholesterol, fasting insulin, and postprandial glucose levels was documented. It is important to note that the oral glucose tolerance test (OGTT) demonstrated a substantial increase in the insulin sensitivity index following 30 min, signifying an enhancement in the functionality of the insulin–glucose axis as a consequence of quercetin therapy [119].

In summary, the protective effects of QE on pancreatic islet β-cells can be classified into three main areas: enhancing insulin secretion by stimulating β-cells to respond to glucose and secretory factors, protecting β-cells from damage (mainly due to antioxidant properties that reduce oxidative stress and promote cell survival), and promoting β-cell proliferation (which may contribute to the renewal of pancreatic islet cell populations and improve long-term pancreatic endocrine function). The results of this study suggest that quercetin may possess significant therapeutic potential when used in conjunction with other treatments for diabetes, through its direct action on β cells and the modulation of insulin secretion.

Insulin resistance is an increasingly prevalent pathological condition in which the body’s cells (predominantly skeletal muscle, adipose tissue, and hepatocytes) fail to respond appropriately to insulin. This results in reduced glucose uptake from the bloodstream and impaired glucose homeostasis, which in the long term leads to the development of hyperglycemia and type 2 diabetes [120]. One of the significant factors that has been identified as a catalyst for the advancement of insulin resistance is chronic exposure to elevated concentrations of glucose and free fatty acids. This exposure has been demonstrated to induce an excessive production of ROS. The accumulation of ROS leads to oxidative stress, tissue damage, and impaired insulin signaling in peripheral metabolic organs. As demonstrated in a study by Babacanoglu [121], long-term hyperglycemia in rats with streptozotocin-induced diabetes results in decreased phosphorylation of the insulin receptor and its substrate, insulin receptor substrate-1; decreased expression of endothelial nitric oxide synthase; and a concomitant increase in the expression of inducible nitric oxide synthase. These changes result in impaired insulin response and exacerbate insulin resistance.

In response to these disorders, QE has demonstrated the capacity to enhance metabolic parameters in diabetic animals. In a study using rats with streptozotocin-induced diabetes, it was demonstrated that oral administration of QE at a dose of 100 mg/kg led to a substantial reduction in glycemic gains between 30 and 180 min following glucose loading. This finding suggests that QE enhances insulin sensitivity and improves tissue glucose utilization. The mechanism may involve the restoration of the balance between endothelial nitric oxide synthase and inducible nitric oxide synthase, which has been demonstrated to reduce nitrosative stress and improve vascular endothelial function [121]. As posited by Zhao et al. [122], the potential for QE to impact signaling pathways associated with glucose metabolism is a salient consideration. In an animal model in which male Wistar rats were fed a high-fat diet for a period of three weeks and then treated with streptozotocin to induce diabetes, the administration of QE resulted in improved insulin sensitivity and reduced hepatic glucose production. This effect was associated with increased expression of the silent information regulator 1 (SIRT1) protein and activation of AMP-dependent protein kinase (AMPK) in the duodenal mucosa, suggesting the involvement of quercetin in the regulation of the gut–liver axis.

In a separate study, Rodríguez and colleagues discovered that even low doses of QE (2.5 mg/kg) could enhance peripheral insulin sensitivity in mice with streptozotocin-induced diabetes. This improvement was associated with the upregulation of SIRT1 expression and downregulation of protein tyrosine-phosphate phosphatase 1B (PTP1B), an enzyme that negatively modulates insulin signaling [123]. Subsequent studies by Zhao et al. evaluated the synergistic effects of QE and acarbose in db/db mice, a model of type 2 diabetes. The dietary supplementation of QE (0.08%) and acarbose (0.03%) resulted in substantial reductions in plasma glucose levels and enhancements in indices of insulin resistance [122]. The molecular mechanisms responsible for this effect included decreased cyclic AMP (cAMP) accumulation, reduced free fatty acid influx, protein kinase A (PKA) activation, the stabilization of phosphodiesterase 3B (PDE3B) activity, and increased diacylglycerol (DAG) accumulation [18].

The accrual of preclinical data indicates that quercetin may have a significant role in counteracting insulin resistance through multiple mechanisms, including the modulation of oxidative stress, the improvement of endothelial function, the activation of SIRT1/AMPK signaling pathways, and the reduction in negative regulators of insulin signaling, such as PTP1B. Whilst these results are encouraging, further studies—especially randomized clinical trials—are required to confirm the efficacy and safety of QE as a potential treatment for the facilitation of insulin secretion and enhancement in insulin sensitivity in humans.

### 5.2. Reduction in Intestinal Glucose Absorption by the Inhibition of α-Glucosidase

The digestion of carbohydrates begins in the mouth and continues in the small intestine. Complex polysaccharides are gradually broken down into simpler sugars, primarily glucose, which are then absorbed into the bloodstream. Key enzymes in this process include α-glucosidase, found in the small intestinal brush border, and α-amylase, which is secreted by the salivary glands and pancreas [62]. Patients with type 2 diabetes experience increased activity of carbohydrate-digesting enzymes, especially after meals. This leads to a sharp rise in blood glucose levels, a phenomenon known as postprandial hyperglycemia. This is one of the most important therapeutic targets in diabetes treatment, as prolonged glycemic fluctuations are associated with increased oxidative stress, inflammation, and the accelerated development of vascular complications. Therefore, inhibiting α-amylase and α-glucosidase activity is considered an effective therapeutic strategy for controlling the increase in blood glucose levels after eating. In this context, QE and its derivatives have attracted particular interest as natural inhibitors of digestive enzymes [124]. Studies by Honghui et al. have demonstrated the strong inhibitory properties of QE and its glycoside derivatives against intestinal α-glucosidase activity in vitro [125].

These studies suggest that quercetin may delay the breakdown of oligosaccharides and disaccharides into glucose, thereby slowing its absorption and reducing the postprandial rise in blood glucose levels. In contrast, modest α-amylase inhibitory activity may be physiologically beneficial, as complete inhibition of this enzyme is often associated with gastrointestinal side effects (e.g., bloating). Therefore, selectively inhibiting α-glucosidase without strongly affecting α-amylase may be a safer therapeutic strategy.

### 5.3. Promotion of Glucose Uptake in Various Tissues

The process of glucose utilization by cells necessitates the transportation of glucose across cell membranes, a process that is facilitated by a family of transport proteins known as glucose transporters (GLUTs). GLUT4, the principal glucose transporter in skeletal muscle cells and adipocytes, fulfills a unique function. Its activity is subject to regulation by insulin signaling, which is initiated through an insulin receptor with tyrosine kinase activity. Subsequent to the binding of insulin to its receptor, a series of intracellular reactions ensues, culminating in GLUT4 translocation from the cytoplasmic vesicles to the plasma membrane surface. This process facilitates augmented glucose uptake by cells. In type 2 diabetes, however, this complex mechanism is disrupted, primarily due to impaired GLUT4 translocation and abnormal insulin signal transduction. Impaired GLUT4 translocation to the cell membrane has been demonstrated to limit the efficient transport of glucose into cells, thereby contributing to the development of insulin resistance [126].

However, AMP-activated protein kinase (AMPK), a pivotal regulator of cellular energy homeostasis, has been demonstrated to stimulate GLUT4 translocation and promote glucose uptake independently of insulin. Hamilton et al. demonstrated that QE, a naturally occurring plant flavonoid, activates glucose uptake in C2C12 skeletal muscle cells through an AMPK-dependent, insulin-independent mechanism [127]. Furthermore, Dai et al. [128] conducted a study that confirmed QE supplementation led to a significant reduction in blood glucose levels and increased GLUT4 expression, enhancing glucose uptake on the surface of skeletal muscle cells via the AMPK pathway. Collectively, these findings indicate that AMPK could be a viable therapeutic target for the treatment of diabetes and its complications. A substantial body of research has demonstrated that quercetin possesses the capacity to enhance glucose uptake in the absence of insulin. This phenomenon may be attributed to the augmented expression of GLUT4 within the cell membrane. Furthermore, it has been demonstrated that QE rapidly induces the translocation of GLUT4 by increasing the expression of estrogen receptor α (ERα), which in turn activates PI3K/Akt or AMPK/Akt signaling cascades. The consequence of this activation is an increase in glucose transport into muscle cells [127]. It has been demonstrated that QE exerts its effects on cell signaling, thereby enhancing glucose utilization. This function is achieved through the modulation of glucose transport and the regulation of the insulin receptor pathway. In doing so, it exhibits effects analogous to those of rosiglitazone, a proliferator-activated receptor gamma (PPARγ) agonist that also improves tissue insulin sensitivity [129]. Animal studies on the antidiabetic effects of quercetin not described in the text are included in Table 2.

### 5.4. Quercetin and Complications of Chronic Hyperglycemia—Role in Organ Protection

Chronic hyperglycemia, a hallmark of untreated or inadequately controlled diabetes, is a pivotal factor in the development of vascular damage, encompassing both large (macroangiopathy) and small (microangiopathy) vessel involvement. These complications include retinopathy, nephropathy, neuropathy, as well as cardiovascular disease. One of the most serious microangiopathic complications of diabetes is diabetic retinopathy, which is a leading cause of blindness and significant deterioration of visual acuity in adult patients with diabetes [141].

Recent studies have indicated that QE may possess therapeutic potential in the treatment of diabetic retinopathy. In a rat model of streptozotocin-induced diabetes, administration of QE at a dose of 150 mg/kg resulted in a significant reduction in the expression of pro-inflammatory and angiogenic factors such as monocyte chemoattractant MCP-1, matrix metalloproteinase MMP-9, and vascular endothelial growth factor. Concurrently, a decline in oxidative stress-induced protein damage was observed, thereby substantiating the substantial antioxidant efficacy of QE in the context of retinal protection [142].

Diabetic nephropathy, another significant complication of long-term hyperglycemia, develops as a result of chronic exposure of renal cells to excess glucose, leading to increased inflammatory processes and fibrosis. This progression is characterized by the activation of multiple molecular mediators, encompassing growth factors, cytokines, and enzymes, which collectively contribute to cell proliferation, renal tissue hypertrophy, and the development of interstitial fibrosis. Quercetin has been demonstrated to be efficacious in the inhibition of these deleterious processes by means of the inactivation of the SphK1-S1P (sphingosine kinase-1 and sphingosine-1-phosphate) signaling pathway, which is implicated in the pathogenesis of renal fibrosis [143]. Another aspect of long-term hyperglycemia is its detrimental effect on the central nervous system, which can result in neurodegenerative complications. Oxidative stress, caused by chronic glucose elevation, has been identified as a potential pathogenetic link in diseases such as Parkinson’s disease and Alzheimer’s disease [144]. A study conducted on rats with streptozotocin-induced diabetes demonstrated that administration of QE resulted in enhanced cognitive function, including memory [145]. This finding suggests that QE may have neuroprotective effects in the context of diabetes.

Macroangiopathies, including but not limited to hypertension, diabetic cardiomyopathy, and ischemic heart disease, are also exacerbated by chronic hyperglycemia and insulin resistance [146]. In a rat model of streptozotocin-induced diabetes, it was demonstrated that QE, administered in isolation or in conjunction with glibenclamide, exhibited a substantial reduction in myocardial damage caused by diabetic cardiomyopathy. This effect was found to be dose-dependent [147]. Furthermore, studies have demonstrated that QE exerts cardioprotective effects by stimulating the expression of endothelial cell receptors and increasing nitric oxide production, thereby enhancing vascular function [148]. Furthermore, a clinical trial involving patients with type 2 diabetes demonstrated that quercetin supplementation could significantly reduce systolic blood pressure, thereby further substantiating its potential in the treatment of cardiovascular complications [149].

A growing body of preclinical research has indicated a multifaceted beneficial effect of QE on the progression of diabetes and its complications. However, the majority of available data originates from studies conducted in animal models. A paucity of clinical studies has been conducted on humans, and only a small number of the mechanisms of action of QE have been thoroughly described and confirmed. The role of iron metabolism abnormalities in patients with diabetes has recently been the focus of considerable attention. Abnormal iron accumulation and distribution have been demonstrated to contribute to increased inflammation and oxidative stress, which in turn can exacerbate the progression of diabetic complications [150]. However, to date, there has been a paucity of studies that have analyzed the potential role of QE in regulating iron metabolism in the context of diabetes. It is therefore evident that further research is required in both basic and clinical contexts. This is to enable a more comprehensive evaluation of the potential of quercetin as a therapeutic agent in the treatment and prevention of retinopathy, nephropathy, neuropathy, and other complications resulting from chronic hyperglycemia.

## 6. Conclusions and Future Perspectives

Quercetin is a naturally occurring flavonoid with potent antioxidant properties, exhibiting a broad spectrum of biological activities, including anti-inflammatory, anticancer, cardioprotective, and neuroprotective effects. In recent years, there has been an increase in research focusing on its potential use in the treatment of diabetes and the prevention of its complications. Scientific reports indicate that the incidence of both diabetes and its metabolic complications may be significantly reduced following the use of QE as a therapeutic intervention.

Numerous preclinical studies have delineated the mechanisms by which quercetin exerts its antidiabetic effects. It has been demonstrated that QE has the capacity to reduce blood glucose levels through a number of complementary mechanisms, including the induction of peroxisome PPARγ expression, the reduction in glucose absorption in the gastrointestinal tract, the enhancement of glucose uptake by peripheral tissues, and the improvement of cellular sensitivity to insulin. Collectively, these mechanisms contribute to enhanced glycemic regulation.

Nevertheless, despite the encouraging biological properties of QE, its clinical use is constrained by its low bioavailability. This is primarily attributable to its low solubility in water and its limited stability within the gastrointestinal environment. Consequently, future research should concentrate on the development of innovative forms of QE administration that will enhance its stability and biological efficacy. Examples of such solutions include the formation of quercetin complexes with macromolecules, the use of nanoemulsion technology, microencapsulation, or combining QE with other bioactive compounds for synergistic effects. A further research direction that merits exploration is a comparison of the efficacy of pure QE with forms of natural plant extracts containing this flavonoid. Such comparisons may provide a broader perspective on the efficacy and potential pharmacokinetic and pharmacodynamic differences between isolated forms and complex plant matrices. Another significant issue that requires further research is determining the optimal QE dosage for use as an adjunctive diabetes treatment, particularly in comparison with existing drugs. In light of the mounting interest among patients in natural therapeutic substances, research into QE may have significant practical and societal implications. In addition, it is imperative to undertake a comprehensive analysis of the impact of QE on carbohydrate metabolism and its effect on the glycemic index of foods, with particular emphasis on those comprising substantial quantities of starch or glucose. It is imperative to comprehend the mechanisms through which quercetin functions in the context of co-digestion, that is to say, when ingested concomitantly with food, and its impact on the glycemic characteristics of specific products under real-world consumption conditions. The development of a new generation of functional foods enriched with QE could be facilitated by such research, for example, in the form of nanoemulsions, starch–quercetin complexes, or other forms that enhance its bioavailability. While in vitro and preclinical studies have yielded valuable data, the confirmation of these results is crucial through in vivo experimentation, employing both animal models and human clinical trials. Mechanistic studies and long-term observations are also required to assess the safety of QE and its long-term metabolic effects.

In conclusion, it is important to note that the effects of QE may vary depending on the specific type of diabetes, whether it is type 1 or type 2. Therefore, further studies are needed to differentiate its effects in the context of different disease pathomechanisms, potential side effects, and interactions with other drugs or bioactive substances. In addition, the effect of QE on various forms of oxidative and inflammatory stress, which play a key role in the pathogenesis of diabetes and its complications, must also be considered.

## Figures and Tables

**Table 1:** Sources of dietary quercetin based on the USDA Database for the Flavonoid Content of Selected Foods [31].

Number	Common Name	Scientific Name	QE Content [mg/100 g]
1	Capers, raw	*Capparis spinosa*	233.84
2	Capers, canned	*Capparis spinosa*	172.55
3	Lovage leaves	*Levisticum officinale*	170
4	Radish leaves	*Raphanus sativus*	70
5	Dill weed, fresh	*Anethum graveolens*	55.15
6	Coriander leaves (cilantro)	*Coriandrum sativum*	53
7	Onion, yellow, raw	*Allium cepa*	39.21
8	Chili pepper, hot, green	*Capsicum annuum*	15
9	Cranberries, raw	*Vaccinium macrocarpon*	14.02
10	Lingonberries	*Vaccinium vitis-idaea*	12
11	Blueberries, raw	*Vaccinium corymbosum*	7.71
12	Buckwheat flour, whole	*Fagopyrum esculentum*	9.03
13	Apple, skin only	*Malus domestica*	19.36
14	Black grapes	*Vitis vinifera*	4.47
15	Black currants	*Ribes nigrum*	3.87
16	Kale, raw	*Brassica oleracea* var. *sabellica*	3.71
17	Chokeberries (Aronia)	*Aronia melanocarpa*	3.8
18	Broccoli, raw	*Brassica oleracea* var. *italica*	3.48
19	Tea, black, brewed	*Camellia sinensis*	2.07
20	Tea, green, brewed	*Camellia sinensis*	2.2
21	Elderberries	*Sambucus nigra*	2.42
22	Rocket (arugula), raw	*Eruca sativa*	2.25
23	Sorrel, raw	*Rumex acetosa*	2.1
24	Apple, Gala, with skin	*Malus domestica*	3.8
25	Chokeberries (Aronia)	*Aronia melanocarpa*	3.8
26	Broccoli, raw	*Brassica oleracea* var. *italica*	3.48
27	Red wine	*Vitis vinifera*	2
28	Tea, black, brewed	*Camellia sinensis*	2.07

**Table 2:** Animal studies on the antidiabetic effects of quercetin.

Animal Model	Dosage	Effect	Ref.
C57BL/6J mice	1.5 mg/kg bw, 4 months	↓ insulin resistance ↓ serum glucose level ↑ insulin intensity per islet cell ↑ protective effect on size and structure of pancreatic β-cells	[130]
STZ at a dose of 55 mg/kg bw, ip, Wistar albino rats	Orally QE 15 mg/kg/day for 28 days	↓ blood glucose ↑ serum insulin level	[131]
STZ at a dose of 55 mg/kg bw, ip, Wistar albino rats	Orally QE 15 mg/kg/day for 28 days	↓ blood glucose ↑ serum insulin level	[132]
i.p. of STZ 90 mg/kg.bw in SD rats	QE 2.5, 5, 10, and 20 mg/kg/day orally for 10 weeks	↓ glucose intolerance ↓ endogenous creatinine clearance rate ↓ postprandial glucose and triglyceride levels	[133]
Streptozotocin (50 mg/kg)-induced type 2 diabetes Albino Wistar rats	50 and 100 mg/kg bw of sertiamarin from *Enicostemma axillare* and QE, 28 days	↓ blood glucose protective effect on size and structure of pancreatic β-cells	[134]
Streptozotocin (65 mg/kg)-induced type 1 diabetes in Wistar rats	30, 60, 120 mg/kg bw, 4 months	no impact on blood glucose level	[135]
Sprague–Dawley rats	1 g in 5 mL water (corn starch–QE complex), oral gavage after fasting for 12–14 h	↓ AUC values for blood glucose levels QE at higher level was more effective	[136]
Streptozotocin (40 mg/kg)-induced Albino Wistar rats	50 mg/kg bw in nanoemulsion form, 21 days	↓ blood glucose protective effect on size and structure of pancreatic β-cells	[137]
Sprague–Dawley rats	0.25 g/mL of QE (1.25%, 2.5%, and 5%) in starch complex, postprandial consumption	↓ blood glucose ↑ blood glucose peak time	[138]
Albino Wistar rats	50 mg/kg bw, 8 weeks	protective effect on size and structure of pancreatic β-cells	[139]
Sprague–Dawley rats	18.75, 37.5, and 75 mg/kg bw, postprandial consumption of sucrose and maltose (0.25 g/mL)	↓ blood glucose ↑ blood glucose peak time	[140]

↑—upregulation; ↓—downregulation; bw—body weight; and AUC—Area Under the Curve.

## Data Availability

Not applicable.

## Abbreviations

6-OHDA	6-hydroxydopamine
AD	Alzheimer’s disease
ADA	American Diabetes Association
AGIs	Alpha-glucosidase inhibitors
AMPK	AMP-activated protein kinase
AUC	Area under the curve
Aβ	β-amyloid
BBB	Blood–brain barrier
CAT	Catalase
COMT	Catechol-O-methyltransferase
COX-2	Cyclooxygenase-2
CVD	Cardiovascular disease
DPP-4	Dipeptidyl peptidase 4
eNOS	Endothelial nitric oxide synthase
Erα	Estrogen receptor α
FcεRI	High-affinity IgE receptor
GIP	Gastric inhibitory peptide
GLP-1	Glucagon-like peptide-1
GLP-1 RAs	Glucagon-like peptide-1 receptor agonists
GLUT	Glucose transporter
GSH-Px	Glutathione peroxidase
HO-1	Heme oxygenase-1
IFN-γ	Interferon alpha
IL	Interleukin
iNOS	Inducible nitric oxide synthase
LDLs	Low-density lipoproteins
MMPs	Matrix metalloproteinases
NO	Nitric oxide
OGTT	Oral glucose tolerance test
ORAC	Oxygen radical absorbance capacity
PD	Parkinson’s disease
PDE3B	Phosphodiesterase 3B
PGD2	Prostaglandin D2
PKA	Protein kinase A
PPARγ	Peroxisome proliferator-activated receptor
QE	Quercetin
ROS	Reactive oxygen species
SA-β-gal	Senescence-associated β-galactosidase
SGLT2	Sodium–glucose cotransporter-2
SIRT1	Sirtuin 1
SOD	Superoxide dismutase
SphK1-S1P	Sphingosine Kinase 1—Sphingosine-1-Phosphate
SULT	Sulfotransferase
TNF-α	Tumor necrosis factor alpha
UGT	Uridine-5-diphospho-glucuronosyltransferase
VEGF	Vascular endothelial growth factor
